# Sensory, Microbiological and Chemical Changes in Vacuum‐Packaged Blue Spotted Emperor (*Lethrinus* sp), Saddletail Snapper (*Lutjanus malabaricus*), Crimson Snapper (*Lutjanus erythropterus*), Barramundi (*Lates calcarifer*) and Atlantic Salmon (*Salmo salar*) Fillets Stored at 4°C

**DOI:** 10.1002/fsn3.309

**Published:** 2015-12-15

**Authors:** Luisa Fernanda Fuentes‐Amaya, Steve Munyard, Judith Fernandez‐Piquer, Janet Howieson

**Affiliations:** ^1^ Centre of Excellence for Science, Seafood and HealthCurtin Health Innovation Research InstituteCurtin University7 Parker PlaceTechnology ParkWestern Australia6102Australia; ^2^ Food Science and TechnologySchool of Public HealthCurtin UniversityKent STBentleyWestern Australia6102Australia; ^3^ Pathwest Laboratory Medicine WAQEII Medical CentreNedlandsWestern Australia6909Australia; ^4^ Tasmanian Institute of AgricultureSchool of Land and FoodUniversity of TasmaniaSandy BayTasmania7001Australia

**Keywords:** Finfish, quality assessment, specific spoilage organisms, spoilage, total viable count

## Abstract

Quality assessment of finfish fillets during storage is important to be able to predict the shelf life of the fresh product during distribution. Microbial, chemical (pH, TMA, and TVB‐N), and sensory (Quality index assessment QIA, Torry scheme) changes in vacuum‐packaged blue‐spotted emperor (*Lethrinus* sp), saddletail (*Lutjanus malabaricus*), crimson snapper (*Lutjanus erythropterus*), barramundi (*Lates calcarifer*), and Atlantic salmon (*Salmo salar*) fillets stored at 4°C were evaluated for 5 days. Microbiological study included evaluation of TVC (total viable counts), total psychrotrophic organisms, and H_2_S‐producing bacteria. Numbers increased during storage time and reached an average of 8.5, 8.5, and 9.2 log_10_ cfu/g, respectively, for the five different fish species. These levels were above accepted microbiological limits for fish fillets. Although the sensory analyses showed a decrease in quality, none of the finfish fillets were considered unacceptable at the end of the storage trial. Chemically, there was a slight pH increase, but trimethylamine (TMA) levels remained low. However, total volatile basic nitrogen (TVB‐N) levels increased over time, reaching levels above 35 mg/100 g for blue spotted emperor, saddletail snapper, and crimson snapper by the end of the storage period. Results show that the deterioration of finfish fillet quality is a complex event of biochemical, sensory, and microbial factors, and multiple analyses may be required to define acceptability.

## Introduction

Quality monitoring of seafood is an important consideration due to the short shelf life of fresh product. The cost of product loss due to spoilage is a big concern for the seafood industry: it is estimated that 30% of landed fish are lost every year due to spoilage as a result of chemical and microbial activity (Baird‐Parker 2000). In Australia, combined wild and finfish aquaculture harvest is an important part of the economy with 113.310 tonnes produced during 2011–12, valued at approximately $451 million (ABARES 2013). Although there are preservation techniques available, there is still a lack of understanding about the factors that could be addressed to optimize the shelf life of fish (Ghaly et al. 2010).

Microbial growth in combination with enzymatic autolysis and oxidation are the major causes of fish spoilage. The bacterial species that contribute to spoilage are known as SSO (Specific Spoilage Organisms). These organisms produce unacceptable flavors, odors, texture, and/or color, thus reducing the quality of the seafood. Typical SSO of fish include *Shewanella*,* Pseudomonas*,* Photobacterium*,* Aeromonas,* and/or *Enterobacteriaceae* (Gram et al. 2002). The SSO found in fish depend on the initial bacterial load, type of processing, preservation methods, and storage conditions. The spoilage of whole cod and sea salmon in ice is explained by the presence of *Shewanella putrefaciens* (Jørgensen and Huss 1989; Hozbor et al. 2006). *Photobacterium phosphoreum* has been associated with spoilage of haddock fillets stored at temperatures in the range of 0–15°C (Olafsdottir et al. 2006), and in spoilage of vacuum‐ or MAP (modified atmosphere packed) cod fillets stored at 0°C (Dalgaard et al. 1993). In the case of Mediterranean fish stored between 0°C and 15°C and MAP Atlantic salmon, *Pseudomonas* spp. have been identified during spoilage (Koutsoumanis and Nychas 1999; Milne and Powell 2014).

The traditional microbiological assessment used to evaluate finfish fillet quality is TVC (total viable count). The media allows the growth of aerobic mesophilic organisms present in the finfish samples and is commonly used as a measure of quality by food safety authorities and retailers. A TVC level greater than 10^7^ cfu/g in fresh fish is considered unacceptable, (ICMSF 1986) while TVC level of 10^6^ cfu/g corresponds to the maximum level set by some Australian supermarkets. However, it is well documented that not the entire bacterial load enumerated using TVC is responsible for spoilage. Several studies have shown that fish with TVC levels above 10^7 ^cfu/g are still acceptable by sensory evaluation. Some examples include haddock fillets stored at 0°C, 7°C, and 15°C (Olafsdottir et al. 2006) and the European sea bass stored in ice (Kyrana and Lougovois 2002).

Specific Spoilage Organisms are present in low quantities initially but as storage progresses, SSO grow faster than the rest of the bacteria present in the fish (Huis In't Veld 1996). Consequently, specific microbial measurements to determine and quantify SSO are more reliable to evaluate accurately the freshness of seafood. Microbiological media such as Iron Agar (IA) and Long and Hammer Agar (LH) have emerged as being more suitable to determine and quantify the presence of SSO responsible for deterioration of fish and seafood (Gram 1992).

Chemical indexes widely used to evaluate fish quality deterioration include TVB‐N (total volatile basic nitrogen) and TMA (trimethylamine). While TVB‐N measures the overall volatile nitrogen present in the fish, TMA focuses on the reduction in TMAO (trimethylamine oxide) by SSO activity (Howgate 2009).

Sensory evaluation is an important method to monitor the effect of storage conditions on the changes in sensory attributes of the tested food. Sensory evaluation methods such as Quality Index Methods (Erkan and Özden 2008; Vaz‐Pires et al. 2008) or Torry Scheme (Kyrana and Lougovois 2002; Olafsdottir et al. 2006) are useful techniques to measure the quality and freshness for different fish species. Given that consumer acceptability is such an important driver for finfish quality, it has been discussed that instrumental methods should be directly correlated with the sensory assessment (Olafsdottir 1997). The aim of this study was to compare and contrast the various techniques used to evaluate fish quality and freshness to understand better the relationship between the different quality evaluation techniques.

Accordingly, the study evaluated the quality changes of five different vacuum‐packed fillets of common finfish species after 1, 3, and 5 days stored at 4°C. Quality evaluation included the use of different microbiological media, and traditional biochemical and sensory quality assessment analyses.

## Materials and Methods

### Fish source, processing, storage conditions, and sampling

The target fish and the experimental period of storage were chosen based on those species most commonly sold as fillets in local Australian supermarkets and fishmongers, and the normal five‐day shelf life accorded to the raw fillet product by the processors. Fish used for the study included blue‐spotted emperor (*Lethrinus* sp.) harvested in Exmouth Gulf, Western Australia (WA) and saddletail snapper (*Lutjanus malabaricus*) and crimson snapper (*Lutjanus erythropterus*) harvested in the Timor Sea and WA. Barramundi (*Lates calcarifer*) from a WA sea cage operation and Atlantic salmon (*Salmo salar*) aquacultured in Tasmania were also assessed. The fish were refrigerated and transported in 25 kg plastic‐covered tubs to the processing plant in Perth, with the exception of the Atlantic salmon, which was transported in polystyrene eskies. Triplicate samples of each finfish were filleted upon arrival, aseptically divided into 6 × 200 g portions, vacuum packed and stored at 4°C ± 1°C. Two random samples of each vacuum‐packed finfish were collected after 1, 3, and 5 days from filleting, and sent to the testing laboratories in a box equipped with ice packs. For each finfish species, one pack was used for microbiology analyses at the Food Microbiology laboratories (L Block, University of Western Australia, Perth) and a second pack was used for sensory assessments at the Food Sensory laboratories (Building 400, Curtin University, Perth). Two independent experimental trials were performed in February and May 2012. Samples from both trials were used for microbiological, chemical, and sensorial assessments. Both trials were performed under the same conditions, with no significant differences noted between them. Accordingly, data from same fish fillet type was combined for analysis against the study objective.

### Microbiological analysis

Duplicate fish fillet samples (10 g) were aseptically weighted and aseptically transferred to sterile plastic bags. Samples were diluted 1:10 with MRD (Maximum Recovery Diluents) (peptone saline water containing 0.1% peptone and 0.85% saline), and homogenized for 1 min at the highest settings in a Seward Stomacher^®^ (model 80, Seward Ltd. West sussex, BN14 8HQ, United Kingdom). Appropriate 10‐fold serial dilutions were prepared in MRD and spiral plated (0.05 mL) in duplicate onto selected media using a Whitley Automated Spiral Plater. TVC agar plates were incubated at 30°C for 48 h and results were expressed as total viable bacteria counts (Buchbinder et al. 1953; Anon 1994). Iron agar (IA) plates were incubated at 25°C for 48 h and used to enumerate hydrogen sulphide‐producing bacteria (Gram et al. 1987; Nordic Committee on Food Analysis, 2006). Long and Hammer agar (LH) (Van Spreekens (1974) and Nordic Committee on Food Analysis (2006)), were prepared and incubated at 15°C for 5 days for quantitative determination of psychotrophic bacteria.

### Chemical analyses

For each analysis, the vacuum package for each fish species was opened and the sample was minced using sterile scissors to an approximate size of 0.5 cm^2^. A portion of 10 g was transferred to a 10 mL glass beaker, then homogenized with 25 mL of 7.5% aq TCA (trichloroacetic acid, Sigma‐Aldrich, Saint Louis, MO 63101, United States). The homogenate mixture was then passed through a Whatman No. 1 filter paper. The filtrates were stored at −20°C until TMA and TVB‐N levels were determined following the procedures described by Baixas–Nogueras and others. (Baixas‐Nogueras et al. 2001). The TMA analysis included a traditional Dyer colorimetric as described by the Association of Official Analytical Chemists (AOAC 1995). Absorbance at 410 nm wavelength was measured using a Konica Minolta Spectrophotometer (CM‐500i/CM‐500C). The TVB‐N test was performed according to the official EU method and involved the use of a Kjeltec System (model 1002 Distilling Unit, Tecator Inc., Boulder, CO). Results were expressed as mg/100 g sample.

The pH of 1:5 diluted samples was measured at room temperature with a glass electrode connected to a Hanna pH meter (Hanna HI 9321, Hanna Instruments Inc, Rhode Island, 02895, United States).

### Sensory analyses

Sensory analyses included in this study had ethics approval from Curtin University (HREC Number RD‐47‐10).

A Quality Index Assessment Scheme (QIA) for the raw fish fillets from each of the target species was developed using previously described procedures for quality assessment of fish (Boulter et al. 2006; Martinsdóttir et al. 2009). Each scheme consisted of six attributes (appearance, transparency, texture, bloodlines, odor, and gaping) and a score system ranking from 0 (high freshness) to 3 (low freshness). For sensory analyses, each fish fillet was placed on a separate plastic tray labeled with the corresponding name of the fish. Every day during the development of the scheme, two trained panelists assessed the fish on sterile, neutral colored desks using the draft list of attributes. Separate species schemes were developed based on this initial assessment. The developed QIA scheme for each species is shown in Table 1.

**Table 1:** Quality index method schemes for sensory evaluation of vacuum‐packaged barramundi, Atlantic salmon, blue‐spotted emperor, saddletail snapper, and crimson snapper fillets stored at 4°C

Fish species	pH			TMA (mg/100 g)			TVB‐N (mg/100 g)		
	1^a^	3	5	1	3	5	1	3	5
Barramundi	6.3	6.5	6.6	0.37	0.30	0.38	27.44	22.96	31.64
Atlantic salmon	6.2	6.3	6.5	0.19	0.28	0.28	20.44	26.88	28.56
Blue‐spotted emperor	6.5	6.6	6.6	0.23	0.39	0.18	25.90	27.16	41.44
Saddletail snapper	6.4	6.5	6.7	0.21	0.40	0.40	22.68	27.30	58.52
Crimson snapper	6.3	6.4	6.6	0.33	0.33	0.43	25.48	30.52	58.10

a Storage time (days).

The Torry Scheme (Martinsdóttir et al. 2009) was used for sensory (odor and flavor) evaluation of the cooked fish fillets. The maximum score is 10 indicating the highest freshness in flavor and odor. An average score of 5.5 is recognized as the minimum acceptable for human consumption (Boulter et al. 2006; Martinsdóttir et al. 2009).

For the experiments, sensory assessments (both QIA on raw fillets and Torry assessment on cooked fillets) were undertaken at the School of Public Health at Curtin University in Western Australia. Seven volunteers formed the sensory panel and as all seven had previously been trained in QIA and Torry scheme, there was need for only one training session at the facilities of the industrial partner. Before participating in the sensory analysis, all the panelists were given a consent form to sign, which described the background to the study and their sensory evaluation schedule.

At each session, each panelist was given a Torry scoresheet, “*Freshness evaluation of cooked lean fish*” on which to record their assessment of the odor and flavor for each of the five types of cooked fish fillets. All fillets were a standardized size of 2 cm × 2 cm and were cooked in a microwave oven (1800 Watts) for 20 sec in the kitchen located behind the assessing area. Each panelist was seated in a separate sensory booth and access to the kitchen was supplied by a small window, which could be opened only from the kitchen side. A random order of presentation of samples was used throughout the assessments, and all panelists assessed a piece of the same fish sample, each sample was placed on a white plate, and labeled with an individual random number. The panelists were provided with water and plain crackers to allow them to assess each sample with a fresh palate.

### Statistical analysis

The five fish fillets in the study from both trials were analyzed under the same conditions and the data for both trials were combined. The data were analyzed using the statistical software SPSS 10.0 for Windows (SPSS Inc., Chicago, IL), and the results were statistically computed, graphed, and tabulated. As the data were not normally distributed, a nonparametric test Kruskal–Wallis test (one‐way ANOVA) with significance level of 0.01 was applied.

## Results and Discussion

### Microbiological analysis

**Figure 1:**
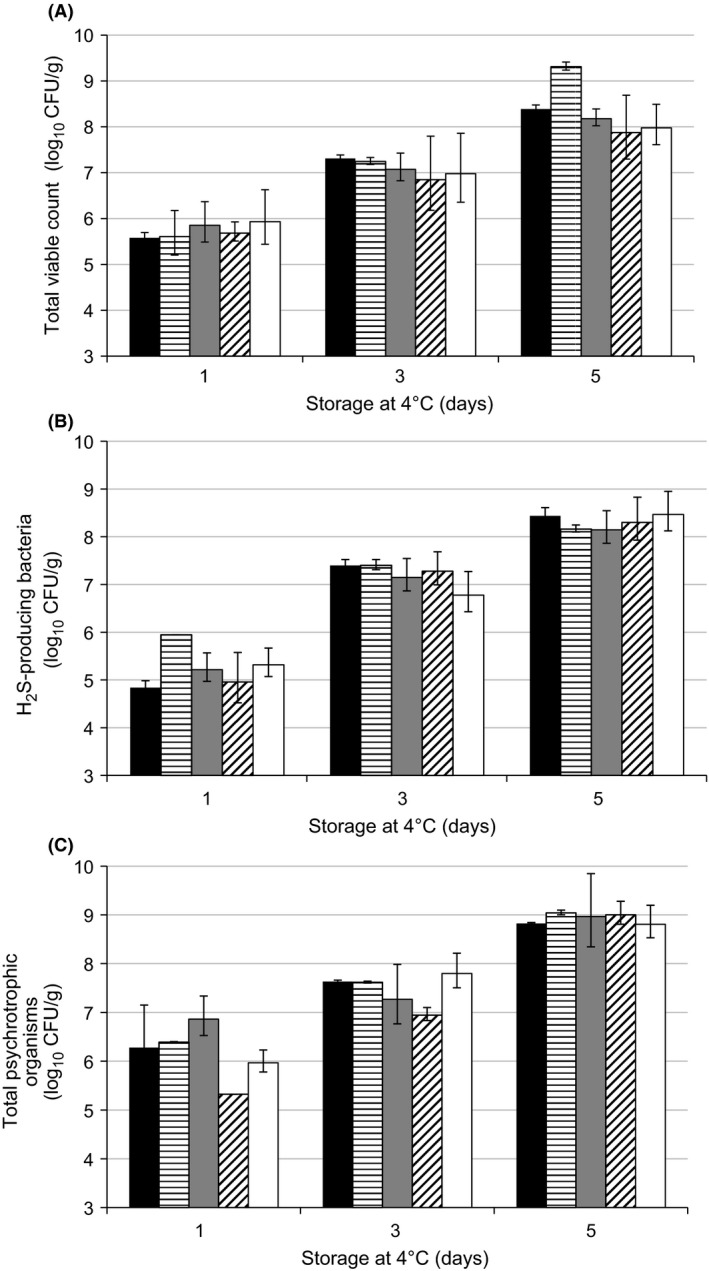
Microbiological changes in vacuum‐packaged barramundi (black), Atlantic salmon (horizontal lines), blue‐spotted emperor (gray), saddletail snapper (diagonal lines), and crimson snapper (white) fillets stored at 4°C. Bars indicate standard deviations.

Changes in microbiological numbers with time in filleted barramundi, Atlantic salmon, blue‐spotted emperor, saddletail snapper, and crimson snapper during storage at 4°C are shown in Figure 1. As observed for cod fillets (Dalgaard et al. 1993), vacuum packaging did not limit bacterial growth. TVC levels were an average of 5.8 ± 0.3 log_10_ cfu/g (Day 1), 7.2 ± 0.5 log_10_ cfu/g (Day 3), and 8.5 ± 0.8 log_10_ cfu/g (Day 5) for the five different fish species (Fig. 1A). Lower initial mesophilic aerobic bacteria, ranging between 2.5 to 4 log_10_ cfu/g, have been previously been reported during microbiological assessment of whole rainbow trout, whole sardines, and sea salmon (Chytiri et al. 2004; Hozbor et al. 2006; Erkan and Özden 2008). Differences in microbiological levels among finfish species may be related to a diverse initial composition depending on the harvest water temperature, handling, and storage situation (Huis In't Veld 1996; Chytiri et al. 2004). In addition, spoilage mechanisms have been observed to be faster in fillets compared to whole fish (Huss 1995; Chytiri et al. 2004). The higher initial TVC levels found in this study may also be because first sampling was performed after 1 day of chilled storage (day 1)., a 10‐fold higher TVC levels was observed for haddock fillets tested after 1 day of cooled storage in Styrofoam boxes compared with fillets processed 1 day postcatch (Olafsdottir et al. 2006). In fact, the TVC levels in all finfish fillets analyzed in this study were found to be above the acceptability limits (6 log_10_ cfu/g) of some Australian supermarkets and close to the microbiological limit for seafood of 7 log_10_ cfu/g (ICMSF 1986) after 3 days.

Other studies have shown longer storage periods to reach these acceptability limits (Kyrana and Lougovois 2002; Chytiri et al. 2004; Hozbor et al. 2006; Erkan and Özden 2008). This difference may be due to the use of lower storage temperatures as it has been observed that the seafood deteriorates twice as fast at 4°C than it does at 0°C (Boulter et al. 2006). Research has shown that TVC growth rates in haddock fillets are found to be approximately three times faster for fish stored at 7°C compared with fish stored at 0°C (Olafsdottir et al. 2006).

For all the species included in this study, TVC on fillets increased more than 2 logs after 5 days at 4°C. Atlantic salmon showed the highest bacterial increase (3.7 logs) and likewise, crimson snapper presented the lowest bacterial increase (2 logs) at the end of the period of study. However, there were no significant difference (*P* > 0.05) found among the five fish fillet species during the entire storage.

Sulphide producers such as *Shewanella putrefaciens* often constitute a major portion of the microbial composition of spoiling fish (Jørgensen and Huss 1989). Sulphide‐producing bacteria levels in the five fish species ranged from 4.8 log_10_ cfu/g to 5.9 log_10_ cfu/g on Day 1 (Fig. 1B). These bacterial levels are higher than the 4 log_10_ cfu/g observed in sardines and sea salmon stored in ice, (Hozbor et al. 2006; Erkan and Özden 2008) but, in the range of the 3–8 log_10_ cfu/g was found in seafood at retail (Gorczyca et al. 1984). As reported in other studies (Huis In't Veld 1996; Kyrana and Lougovois 2002), H_2_S‐producing bacteria levels are lower than TVC levels at the beginning of storage, but levels become less different after a few days of storage. A similar bacterial growth rate pattern was observed for each of the different finfish species in this study and the average of hydrogen sulphide‐producing bacteria levels were 7.3 ± 0.2 log_10_ cfu/g and 8.5 ± 0.1 log_10_ cfu/g after 3 and 5 days, respectively.

The use of sulphide‐producing bacteria as a spoilage indicator has been found as suitable for freshwater Rohu fish stored at ambient temperature, (Madhusudana Rao and Imam Khasim 2009) but not for sea bass stored in melting ice (Kyrana and Lougovois 2002). Different SSO are able to grow in diverse fish in either synergism or antagonism and produce different spoilage indicators or metabolites (Huss 1995). Although, H_2_S‐producing bacteria and TVC levels were higher in Atlantic salmon, there was no significant difference (*P* > 0.05) among the five fish fillet species and storage time, however, a positive correlation (*R* ^2^ = 0.93) was observed. Thereby, the five different finfish species showed similar microbial deterioration during storage when evaluated with TVC or IA media.

Psychrotrophic bacteria levels were generally higher than TVC or H_2_S‐producer bacteria levels (Fig. 1C). The average levels of psychrotrophic bacteria were 6.1 ± 0.4 log_10_ at Day 1, 7.4 ± 0.2 log_10_ at Day 3, and 9.2 ± 0.3 log_10_ cfu/g after 5 days' storage. Compared with the TVC results, psychrotrophic bacteria counts were 0.5–1.0 log higher. Similar results have been found in sea salmon studies (Hozbor et al. 2006) and therefore, highlight the importance of these cold tolerant organisms in the product's shelf life.

However, the use of TVC as an effective indicator of fish fillet spoilage is uncertain (Gram 1992; Dalgaard 2000). Our study supports the use of media such as LH in combination with low temperature incubation to enable better recovery of the bacteria found in finfish fillets stored at low temperature.

### Chemical analyses

Total volatile basic nitrogen measures the content of ammonia, TMA, and DMA (dimethylamine) (Howgate 2009) in fish. The TVB‐N levels in the five finfish species were in the range of 20.44–27.44 mg/100 mg initially and increased to 28.56–58.52 mg/100 mg after 5‐days storage at 4°C (Table 2). Previous studies classified TVB‐N levels of 10 mg/100 or lower for fresh fish, 20–30 mg/100 g for beginning of spoilage, and above 30 mg/100 g for spoiled fish (Kimura and Kiamukura 1934). Moreover, it has been suggested that the quality classification of fish and fish products based on TVB‐N as “high quality” up to 25 mg/100 g, “good quality” up to 30 mg/100 g, “limit of acceptability” up to 35 mg/100 g, and “spoilt” above 35 mg/100 g (Gulsun et al. 2009). EU document 2074/2005 established limits of acceptability between 25 mg/100 g to 35 mg/100 g. However and possibly being predominantly Australasian species, of the species studied here, only the Atlantic salmon is included in the EU documents in which 35 mg/100 g is the limit of acceptability. TVB‐N values above the highest EU limit of acceptability were found for blue‐spotted emperor, saddletail snapper, and crimson snapper after 5‐days storage at 4°C. Of the five varieties, studied saddletail snapper and crimson snapper presented the highest concentrations (58.52 and 58.10 TVB‐N mg/100 mg, respectively) by Day 5.

**Table 2:** Chemical changes in vacuum‐packaged barramundi, Atlantic salmon, blue‐spotted emperor, saddletail snapper, and crimson snapper fillets stored at 4°C

		Atlantic salmon		Barramundi		Blue‐spotted emperor		Crimson snapper		Saddletail snapper	
Quality parameter		Description	Score	Description	Score	Description	Score	Description	Score	Description	Score
Skin	Brightness	Bright color sheen	0	Gray and pink	0	Pink tinge	0	Bright sheen	0	Creamy pink, bright sheen	0
		Bright color dull sheen	1	Slightly faded	1	Fading	1	Dull	1	Dull pink	1
		Fading color	2	Faded	2	No color	2	Faded	2	Faded	2
		Pale/brown color appearing	3								
Appearance	Transparency	Translucent	0	Translucent	0	Translucent	0	Translucent	0	Translucent	0
		Opaque	1	Opaque	1	Opaque	1	Opaque	1	Opaque	1
Flesh	Texture	Firm and springy	0	Firm and springy	0	Firm elastic	0	Firm and springy	0	Firm and springy	0
		Soft and springy	1	Fingermark remains	1	Soft elastic	1	Soft	1	Soft	1
		Fingermark remains	2			Fingermark remains	2	Mushy	2	Mushy	2
		Mushy	3								
	Blood	Bright red	0	Red	0	Bright red	0	Bright red	0	Bright red	0
		Dark brown	1	Orange	1	Brown	1	Orange	1	Orange	1
				Brown	2			Brown	2	Brown	2
	Odor	Fresh seawater/seaweed	0	Fresh seawater/seaweed	0	Fresh seawater/seaweed	0	Fresh seawater/seaweed	0	Fresh seawater/seaweed	0
		Neutral	1	Neutral	1	Neutral	1	Neutral	1	Neutral	1
		Fishy/metallic	2	Sour	2	Fishy	2	Acidic/fishy	2	Acidic/fishy	2
		Sour	3			Acidic	3	Sour	3	Sour	3
	Gaping	None	0	No gaping	0	No gaping	0	None	0	None	0
		Slight gaping	1	Slight gaping	1	Gaping present	1	Gaping present	1	Gaping present	1
		Significant gaping	2	Significant gaping	2						
Quality Index			13		10		10		11		11

Trimethylamine ranged between 0.19–0.37 mg/100 g at the beginning of the experiments and reached 0.18–0.43 TMA mg/100 mg by Day five. In previous studies, the average concentration for a freshly caught fish is 2 mg TMA‐N/100 g wet weight; however, it can range between 1 and 4 mg/100 g (Oehlenschläger 1997). The level of sensory rejection is variable among species; nevertheless it estimated that between 10 and 15 mg TMA‐N/100 g in aerobic stored fish, and 30 mg TMA‐N/100 g in packed cod (Dalgaard et al. 1993).

These results therefore agree with previous reports that suggest that the TMA indicator is not suitable for the early stages of deterioration. Olafsdottir (1997) discussed the fact that volatile amines such as TMA are present in fresh fish (immediately after capture), but in very low levels and accumulate in the later stages of conservation, depending on the species, temperature, and hygiene. Moreover, TMAO can be first reduced to TMA by endogenous bacteria and later by the action of gram‐negative bacteria as reported by Gram and Huss (1996). Therefore, the high concentration of TMA has been considered mainly as an indicator of spoilage of fish in an advanced state of deterioration.

A slow increase in TMA was also observed in the European sea bass (Kyrana and Lougovois 2002), filleted trout (Chytiri et al. 2004), and Mediterranean fish (Koutsoumanis and Nychas 1999), and the authors suggested that the specific bacterial composition could have influenced the TMA production. Although *S. putrefaciens* is a TMA producer, other spoilage organisms such as *Pseudomonas* spp. do not produce TMA (Gram 1992). In addition, the potential presence of *Pseudomonas* spp. can inhibit the growth of *S. putrefaciens* (Gram and Melchiorsen 1996) causing a reduction below the densities (8–9 log_10_ cfu/g) required for TMA production (Dalgaard et al. 1993; Gram and Huss 1996).

In this study, concentrations of TVB‐N in the fish fillet species showed a weak correlation with concentrations of TMA (*R* ^2^ = 0.450). In general, the fish fillets showed a moderate production of TVB‐N throughout the storage time, while TMA levels always remained low. A similar trend has been observed during other fish quality assessments. Low levels of TMA and an increase in TVB‐N up to 25 mg/100 g was observed in the European sea bass at the end of shelf life, after 19‐days storage in ice (Kyrana and Lougovois 2002). It has been suggested that the increase detected by TVB‐N without the same impact on TMA can be due to ammonia resulting from deamination of amino acids, rather than bacterial activity (Stohr et al. 2001).

The pH value for the five fish species ranged from 6.2 to 6.5 at the beginning of the experiments. The pH values are in agreement with the reported postmortem pH range of 6.0–6.8 (Howgate 2009). There was a slight increase in pH over storage, but changes in the pH values between the f*i*sh species during the entire storage trial were not statistically significant (*P* > 0.05). Minimal changes in the pH values during spoilage have also been observed for trout (Chytiri et al. 2004), in the first half of the storage life of the European sea bass (Kyrana and Lougovois 2002), sardines (Erkan and Özden 2008), haddock fillets (Olafsdottir et al. 2006), and MAP cod fillets (Dalgaard et al. 1993).

It has been described that during spoilage the pH may increase because of production of volatile bases (NH_3_ and TMA), due to SSO action(Fraser and Sumar 1998). However, because of variability in pH starting point among different species, season, and other factors, pH level is not always a good predictor of spoilage (Church 1998). For instance, Pacific coast fish has initial pH value slightly lower than 7, important microbial spoilage could be present with small rises in pH. In contrast to some salmon species, in which the ultimate pH is 6.2, it has been found that spoilage was present with different pH levels (Tarr 1954).

### Sensorial analysis

**Figure 2:**
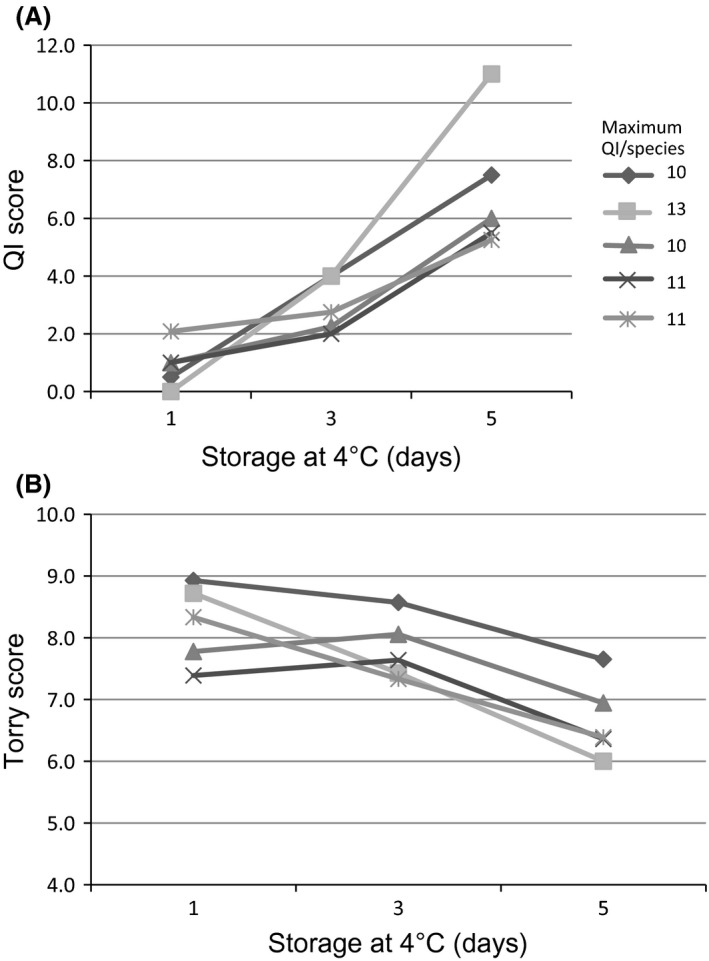
Sensorial changes in vacuum‐packaged barramundi (♦), Atlantic salmon (■), blue spotted emperor (▲), saddletail snapper (×), and crimson snapper (*) fillets stored at 4°C.

Changes in the sensory attributes of the five finfish species fillets during storage at 4°C were evaluated using the QIA method summarized in Table 1. All fish fillets showed an increase in QI demerit points throughout the period of storage as shown in Figure 2A. At Day 1, the lowest QI score (0) was for Atlantic salmon and the highest QI score (2.5) was for crimson snapper (Fig. 2A). After 5‐days storage, Atlantic salmon showed the highest QI score (11) followed by barramundi with a QI score of 7.5. The rest of the species presented a QI score between 5 and 6. This demonstrates different rates of quality deterioration between the different fish species as previously reported (Boulter et al. 2006; Chytiri et al. 2004; Huis In't Veld 1996).

The Torry scheme was used to evaluate the cooked fish and all fish fillets showed a decrease in the Torry score with time as shown in Figure 2B. High negative correlation (*R* ^2^ > 0.93) was observed between the two components evaluated (flavor and odor) in fillets of barramundi, Atlantic salmon, and crimson snapper (*R* ^2^ = 0.94, 0.99 and 0.99; respectively) (Fig. 2B). By contrast, only a moderate correlation (*R* ^2^ = 0.51–0.58) between these parameters was observed in blue‐spotted emperor and saddletail snapper. The accepted level for rejection (average of 5.5) was not reached by the end of the 5‐days storage period for any of the fish species evaluated. Atlantic salmon, had the lowest average score (6) at the end of storage period, which is consistent with the results from the QIA.

Torry assessment as an indicator of freshness quality over time in general shows a clear inverse correlation with the QIA tool: QI score = −1.127 × (days at 4°C) + 9.8595 (*R* ^2^ = 0.95). The results show that using the Torry assessment criteria, all the samples were still suitable for consumption at Day 5.

Cooked fish flavor is considered to be an excellent indicator predictor of freshness quality and shelf life (Lougovois et al. 2002). Our study showed differences in the eating quality with storage time between species. This might have been due to a variation in the size and age of fish used (Pedrosa‐Menabrito and Regenstein 1990) or potential differences in the microbial flora. Further work would be required in this area.

The Torry scheme is a recognized method to evaluate the quality of fish (spoilage related to presence of off‐odors and off‐flavors), and it proved to be beneficial and accurate in this study. In addition, it is a rapid and noninvasive method, which can be performed, with adequate training, in situ. The raw fillet quality assessment measures used during this study, once validated using the quality index established methodologies (Boulter et al. 2006; Martinsdóttir et al. 2009) may be an alternative quality measure as they were correlated well with Torry and microbiological measures.

### Correlation between microbiological, sensory, and chemical analysis

**Figure 3:**
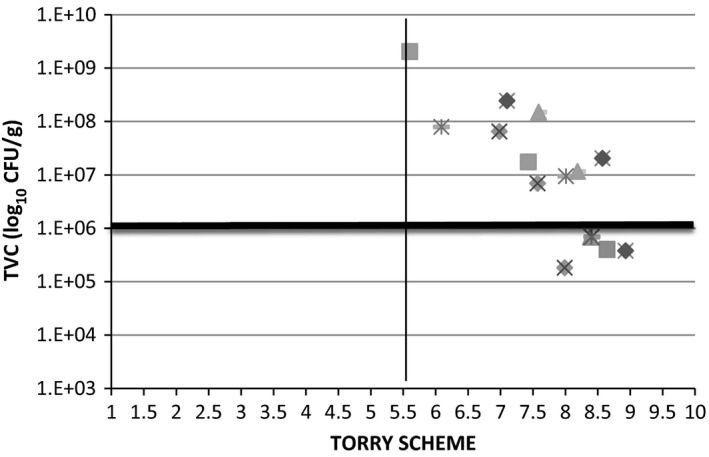
Torry Scheme and Total Viable Count relationship for vacuum‐packaged barramundi (♦), Atlantic salmon (■), blue‐spotted emperor (▲), saddletail snapper (×), and crimson snapper (*) fillets stored at 4°C.

Sensory evaluation methods such as QIA and Torry Scheme have been used to evaluate the shelf life of fish (Erkan and Özden 2008; Kyrana and Lougovois 2002; Olafsdottir et al. 2006; Vaz‐Pires et al. 2008). They are highly accurate and efficient to assess fish freshness, and closely related to consumers criteria. After 3‐days storage at 4°C, fish fillet samples were above the reference limit (>10^6^ cfu/g) and considered unacceptable from the microbial point of view. However, they were still acceptable by sensory panelist perception (Torry scores >5.5). In addition, although microbiological counts were in the range of approximately 8–9 log_10_ CFU/g after 5 days, all fish fillets species were still acceptable by sensory analysis (QIA and Torry scheme level >5.5). This contrast in acceptability based on different assessment criteria (TVC and Torry) is shown in Figure 3. There was a significant negative correlation between TVC and Torry scheme (*r* = −0.719 and *P* < 0.001) Sensory acceptance of seafood above the microbiological reference limit (10^6 ^cfu/g) have also been observed for other fish species (Kyrana and Lougovois 2002; Hozbor et al. 2006; Olafsdottir et al. 2006).

**Figure 4:**
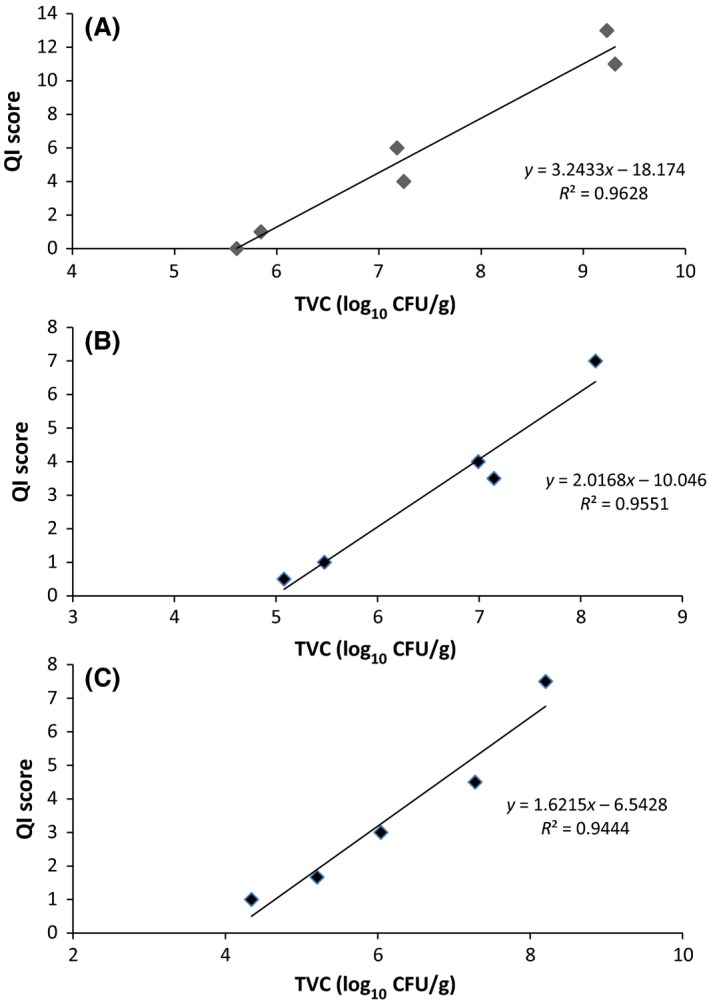
Quality Index Method and Total Viable Count relationship for vacuum‐packaged (A) Atlantic salmon, (B) saddletail snapper, and (C) crimson snapper fillets stored at 4°C.

The different microbiological media used for fish fillets assessment were plotted against QIA scores. TVC provided good correlation with QI score for Atlantic salmon (*R* ^2^ = 0.96), saddletail snapper (*R* ^2^ = 0.96), and crimson snapper (*R* ^2^ = 0.94) (Fig. 4).

Trimethylamine is a useful quality index for microbial spoilage of vacuum‐packed cod fillets (Dalgaard et al. 1993). The fact that TMA did not increase during the overall storage would suggest that TMA as an indicator is not suitable for the early stages of deterioration in Atlantic salmon, barramundi, blue‐spotted emperor, saddletail snapper, and crimson snapper fillets. Similarly, TMA has previously shown little value during spoilage evaluation of cod and ocean perch fillets (Magnusson and Martinsdottir 1995). In the case of TVB‐N, levels above the maximum limit (35 mg/100 g) indicated by the EU were only observed for blue‐spotted emperor, saddletail snapper, and crimson snapper after 5‐days storage at 4°C. However, it was not find statistically significant differences in microbial counts among fish species on Day 5 to justify the TVB‐N differences observed for saddletail snapper and crimson snapper. The result of this study suggest that microbiological and sensory assessment are better indicators of early deterioration of the five fish species tested than chemical indexes such as TVB‐N, TMA, and pH. Freshness deterioration is a complex event of biochemical and microbial interactions. For this reason, further investigation of the bacterial and chemical composition of the different fish fillets species would help to understand better the quality deterioration.

## Conclusions

This study evaluated the effectiveness of quality measurements to determine freshness in vacuum‐packed fillets of five finfish species. The sensory evaluation has shown that all fish fillets in study were still acceptable at the end of the period of study, although microbiological analysis (TVC, H_2_S‐producing organisms, and Total psychrotrophic organisms) showed an exponential bacterial growth throughout all chilled storage period reaching unacceptable levels by day 3. Only Atlantic salmon showed higher microbial counts, whereas the other four varieties did not show significant difference between growths. The observed microbial growth did not have a significant impact on the changes in pH and TMA production. TVB‐N levels increased differently for each fish species. To determine the quality and loss of freshness, it is important to combine quality attributes and correlate them with sensory evaluation, which it is considered the most important method to monitor the effect of storage conditions on fish freshness.

## Conflict of Interest

None declared.
